# Interhemispheric vs. stimulus-response spatial compatibility effects in bimanual reaction times to lateralized visual stimuli

**DOI:** 10.3389/fpsyg.2013.00362

**Published:** 2013-06-19

**Authors:** Antonello Pellicano, Valeria Barna, Roberto Nicoletti, Sandro Rubichi, Carlo A. Marzi

**Affiliations:** ^1^Division for Clinical and Cognitive Neurosciences, Department of Neurology Medical Faculty, RWTH Aachen UniversityAachen, Germany; ^2^Università di PadovaPadova, Italy; ^3^Dipartimento di Filosofia e Comunicazione, Università di BolognaBologna, Italy; ^4^Dipartimento di Comunicazione e Economia, Università di Modena e Reggio EmiliaReggio Emilia, Italy; ^5^Dipartimento di Scienze Neurologiche e del Movimento, Università di VeronaVerona, Italy; ^6^Istituto Nazionale di NeuroscienzeVerona, Italy

**Keywords:** interhemispheric transmission, stimulus-response spatial compatibility, poffenberger paradigm, crossed-uncrossed difference (CUD), corpus callosum, left handedness

## Abstract

In the present study, we tested right- and left-handed participants in a Poffenberger paradigm with bimanual responses and hands either in an anatomical or in a left-right inverted posture. We observed a significant positive crossed-uncrossed difference (CUD) in RTs for both manual dominance groups and both response postures. These results rule out an explanation of the CUD in terms of stimulus-response spatial compatibility (SRSC) and provide convincing evidence on the important role of interhemispheric callosal transfer in bimanual responding in right- as well as left-handed individuals.

## Introduction

A cross-talk between the cerebral hemispheres is essential for integrating perception and motor control between the two sides of the body. The corpus callosum (CC) provides much of the interhemispheric connections enabling this integration. Poffenberger (1912) was the first to tackle this issue experimentally by using a simple reaction time (RT) paradigm to measure interhemispheric transfer time. His rationale relied on the lateralized hemispheric representation of right and left visual hemifields and the lateralized control of distal movement. According to Poffenberger's “anatomical model” when using the hand on the same side of a lateralized visual input stimulus detection and motor response can be integrated within one and the same hemisphere (*uncrossed pathway*). In contrast, when using the hand contralateral to the side of stimulus presentation detection and response must be integrated across hemispheres through the CC (*crossed pathway*). This longer route should result in a slower RT and this is what Poffenberger (1912) and many others since then have found (see for reviews Bashore, 1981; Marzi et al., 1991; Zaidel and Iacoboni, 2003). Since Poffenberger's pioneering study the RT difference between crossed and uncrossed conditions (CUD) is taken as a measure of interhemispheric transfer time (normal values about 3–4 ms). Clear evidence for this “anatomical” callosal interpretation of the CUD comes from its dramatic lengthening following surgical or genetic absence of the CC with values that show at least a 10-fold increase following total callosotomy (Zaidel and Iacoboni, 2003). However, the “anatomical” model has been criticized by various authors on several grounds (see Kinsbourne, 2003; Saron et al., 2003a,b). The criticism that we have considered in the present study is the one originally put forward by Broadbent (1974) which was inspired by the seminal experiments of Wallace (1971) on stimulus-response spatial compatibility (SRSC) effects (see also Umiltà and Nicoletti, 1990; Proctor and Vu, 2006). Broadbent argued that the CUD might be explained in terms of SRSC effects which have higher-level, cognitive instead of lower-level, anatomic determinants. It should be pointed out that in a typical SRSC task a choice rather than a simple reaction paradigm is employed and participants are to discriminate a visual stimulus randomly presented on the left or on the right by pressing a left or a right button. In one block of trials they are instructed to respond with the hand ipsilateral to the stimulus (compatible mapping condition), whereas in the other block they are instructed to respond with the hand contralateral to the stimulus (incompatible mapping condition). Performance is faster in the compatible (same stimulus and response side) compared to the incompatible (opposite stimulus and response side) conditions.

SRSC effects are typically attributed to response selection processes. More recent studies have stated that only if stimulus and response set *overlap* (Kornblum et al., 1990; Kornblum, 1992), that is, share levels of similarities, as is the case for left-right stimuli and responses, the spatial code of the stimulus produces *automatic activation* of the ipsilateral response (see also De Jong et al., 1994). In the compatible mapping condition, the automatically-activated response is identical to the one that was assigned to that stimulus by the instructions. In contrast, with incompatible mapping the required response is the opposite of the automatically-activated one. Thus, when the stimulus is presented the ipsilateral response is automatically activated regardless of whether subjects were instructed to respond with the compatible or incompatible spatial mapping. Simultaneous with this activation is the *response identification* process which is performed through the application of a rule. In the case of compatible mapping response identification proceeds by the simplest and fastest *identity rule* (i.e., “select the response having identical value to stimulus”). Because the automatically-activated and the rule-based response are the same, and this response has been preprogrammed, it can be executed rapidly. Instead, in the case of incompatible mapping response identification is carried out through an *opposite rule* (i.e., “select the response having opposite value to stimulus”). In this case, the verification process will be delayed and response identification will take longer than compatible mapping. Moreover, since the automatically activated and the correct rule-based response differ, the first must be inhibited to avoid conflict with the second at the time of execution. The abort process needed to minimize errors constitutes a second source of delay.

The cognitive bases of SRSC effects are demonstrated when participants are required to cross their hands in that the SRSC effect reverses: responses given with the right hand pressing the left button are slower when the stimulus is on the right compared to when is on the left, while the opposite is true for the left hand. Therefore, crossing the hands in a SRSC RT task yields slower performance for the hand anatomically ipsilateral but spatially contralateral to the stimulus. This finding demonstrates that in a choice RT task, with spatially overlapping responses to visual stimuli, response alternatives are coded as a function of the spatial location of the response devices (e.g., buttons) independent from the anatomical state of the effectors. The SRSC account of the CUD was put to an experimental test independently by Anzola et al. (1977) and by Berlucchi et al. (1977) who demonstrated that in a typical Poffenberger paradigm, i.e., employing simple RT, a CUD effect is still present when participants responded with their hands crossed. When responses were executed with the left hand in the right hemispace and the right hand in the left hemispace, participants were still faster with the hand *anatomically* ipsilateral, but *spatially* contralateral, to the visual stimulus. This rules out an explanation of the CUD in terms of SRSC effects at least for simple RT while they might play an important role in choice RT paradigms (see Berlucchi et al., 1977). In a further experiment using a go-nogo paradigm Berlucchi et al. (1977) found a similar “anatomical” effect as with simple RT.

One should consider, however, that so far the evidence for an anatomical explanation of the CUD has been provided only with unimanual responses and in principle one might argue that SRSC effects might play a role with bimanual responses, a condition in which the importance of interhemispheric transfer may be minimized (for a discussion, see Di Stefano et al., 1980). Therefore, the present study investigated the presence of anatomical vs. SRSC effects in a Poffenberger paradigm with bimanual RT to lateralized stimuli. The presence of an anatomical CUD with bimanual responding would considerably strengthen the callosal relay hypothesis. In a previous study, Di Stefano et al. (1980) assessed the presence of a CUD in unilateral and bilateral key-pressing and lever-pulling conditions with hands in anatomical position. While the unilateral conditions provided significant CUD effects, when bilateral key-pressing and lever-pulling responses were employed, a reliable, albeit small, CUD was present only for key pressing (with the right hand), that is, with a distal response, while was absent for lever pulling, that is, with a proximal response. The authors explained their results by assuming that while unilateral and bilateral distal responses are produced by a lateralized motor pathway, bilateral proximal responses are dependent on a bilateral motor system which ensures a yoked movement of both limbs and therefore no interhemispheric transfer is necessary. However, an important demonstration of the role of the CC with bimanual responses in the Poffenberger paradigm comes from work of Aglioti et al. (1993) who found a lengthening of the CUD following total section or agenesis of the CC for bilaterally executed distal movements. Furthermore, more recently, an increase of the CUD was found with bimanual responses by Ouimet et al. (2010), in total callosum-sectioned patients.

As mentioned above, what is still lacking is evidence on the role of SRSC vs. callosal relay factors for the CUD in a bimanual Poffenberger paradigm. Confirming the results of Anzola et al. (1977) and Berlucchi et al. (1977) with uncrossed as well as crossed posture of the arms but using bimanual responding would provide convincing evidence on the role of interhemispheric transfer in the CUD effect. Moreover, in the present study we wanted to study the role of handedness, that is, a structural variable which might affect interhemispheric transfer. Evidence on the CUD in left-handers is not very abundant: in Marzi et al.'s (1991) meta-analysis were included five studies in left-handers with normal hand posture in writing with a total of 84 subjects and a mean CUD of +4.0 ms that is similar to that of right-handers. In contrast, analysis of four studies of left-handers with inverted hand posture with a total of 77 subjects yielded a mean CUD of −2.4 ms. This suggests that paradoxically in the latter group the crossed pathway might be faster than the uncrossed one perhaps as a result of a more efficient callosal transmission.

Finally, another aim of the present study was to investigate whether an asymmetry of the CUD, which has been found for unimanual responses (for a review see Marzi, 2010) is also present when a bimanual response is employed. Marzi et al. (1991) originally found that in the two crossed hand-hemifield conditions, the left visual field/right hand condition (LVF-RH) yielded faster RT than the right visual field/left hand condition (RVF-LH). Thus, while for the right hemisphere the time to access either hand is roughly similar (CUD = 2 ms), for the left hemisphere it takes almost three times longer to access the left than the right hand (CUD = 5.8 ms). In other words, callosal transfer from the right to the left hemisphere is faster than from the left to right. Interestingly, this asymmetry is reduced or absent in left-handers with either normal or inverted writing hand posture (Marzi, 2010).

## Experiment 1

Experiment 1 essentially replicated the distal bilateral key-pressing condition of Di Stefano et al.'s (1980). Half the participants was to press with each hand the button on the ipsilateral side of space whereas the other half pressed with each hand the button on the contralateral side, while keeping the arms crossed.

### Materials and methods

#### Participants

Twenty-eight students (26 from the University of Bologna and 2 from RWTH Aachen University, 23 females and 5 males, mean age = 21, SD = 3.43) were tested individually. They were all right-handed (72/100, SD = 18.75) as assessed with the Edinburgh Handedness Inventory (Oldfield, 1971). All had normal or corrected-to-normal vision and were naïve as to the purpose of the study.

#### Apparatus and stimuli

The experiment was carried out in a dimly lit and noiseless room. The participants were seated facing a 17 in. screen driven by a 700 MHz PC with the head positioned in an adjustable head-and-chin rest so that the eye distance from the screen was 52 cm. Stimulus presentation and response recording were controlled by the E-Prime Version 1.1 software (www.pstnet.com; Psychology Software Tools, Inc.).

An 8 × 8 mm white fixation cross (0.9 × 0.9° of visual angle) was presented on a black background at the beginning of the experiment. The stimulus was an 18 × 18 mm (2 × 2°) light gray square presented 15° to the left or right of the fixation cross. Two button boxes were aligned with the left and right stimulus locations, respectively and connected to a PST serial response box.

#### Procedure

The fixation cross remained visible across the experiment and a tone signaled the start of each trial. After a 1000–1800 ms random interval the stimulus was presented for 100 ms and then followed by a 1000 ms blank during response collection. Participants were instructed to press the left and the right button *simultaneously* when the stimulus appeared on either side of the screen. Half the participants (*n* = 14) pressed the left and the right button with the left and the right index finger, respectively (*anatomical condition*). For the other half, the position of the hands was crossed at mid-forearm with respect to the response buttons. Thus, participants were instructed to press the left and the right button with the right and the left index finger, respectively (*inverted condition*). Furthermore, in the first half of the experiment, half participants had their hands crossed with left forearm placed over the right, while in the second half they switched to the opposite arrangement. The other half of participants followed the opposite order of forearm arrangements.

The location of the visual stimuli and of the response buttons were irrelevant to the task; both ipsilateral and contralateral RTs were collected on each trial. Omissions, single button presses and anticipations (key presses before or within stimulus onset) were considered errors and discarded. After a correct response, the RT of the first pressed button was displayed for 600 ms, otherwise, error messages were displayed for 1200 ms. The experiment consisted of one practice block of 20 trials followed by four experimental blocks of 100 trials each separated by a rest break. Response omissions (0.4%), unimanual responses (1.2%), responses faster than 120 ms (0.4%) and slower than 700 ms (0.2%) were not considered for statistical analysis.

### Results

**Figure 1:**
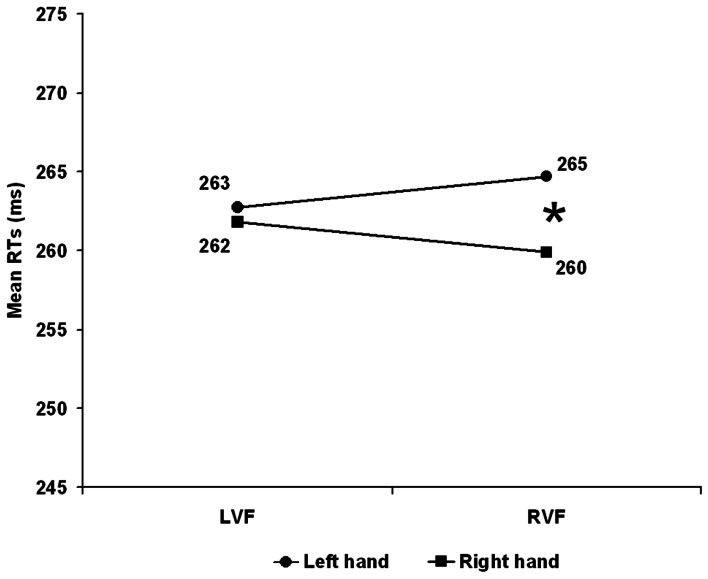
**Experiment 1: Right Handers**. Mean reaction time (RT) as a function of visual hemifield of stimulus presentation and response hand. LVF, left visual field; RVF, right visual field. The asterisk indicates significant *post-hoc* test (*p* < 0.025).

Correct RTs^1^ were submitted to a mixed ANOVA with *Hand arrangement* (anatomical vs. inverted) as between-participants and *Visual field* (Left vs. Right) and *Responding hand* (Left vs. Right) as within-participants factors. Paired sample *T*-tests were employed as *post-hoc* tests; Bonferroni correction was applied so that the *p*-level was decreased to 0.025 for the first order interactions. All main effects were far from significance. *Hand arrangement*: *F*_(1, 26)_ < 1, *p* = 0.422. *Visual Field: F*_(1, 26)_ < 1, *p* = 0.990; *Hand*: *F*_(1, 26)_ = 2.346, *p* = 0.138. The interaction *Visual Field* × *Hand arrangement* was not significant: *F*_(1, 26)_ = 1.121, *p* = 0.229, while, the *Hand × Hand Arrangement* interaction was marginally significant *F*_(1, 26)_ = 4.116, *p* = 0.053 with the right hand slightly faster (254 ms) than the left hand (261 ms) with the inverted, but not with the anatomical arrangement (left hand = 267 vs. right hand = 268 ms). Importantly, the *Visual Field* × *Hand* interaction was significant *F*_(1, 26)_ = 20.532, *p* < 0.001 witnessing the presence of an overall CUD of +2.0 ms, see Figure 1. When the stimulus was in the RVF the right hand responded faster than the left hand (260 vs. 265 ms) *t*_(27)_ = 2.454, *p* = 0.021 whereas, when the stimulus was in the LVF there was no difference between the hands (262 vs. 263 ms) *t*_(27)_ = 0.462, *p* = 0.648. The important finding here was that these effects were independent from hand arrangement as shown by the non-significant second order *Hand Arrangement* × *Visual Field* × *Hand* interaction *F*_(1, 26)_ = 1.028, *p* = 0.320.

Thus, by ruling out the role of SRSC, this result extended the anatomical account to a CUD obtained with bimanual responding in a population of right handers. Interestingly, the CUD was asymmetric with a significant 5 ms CUD when the stimulus was presented on the right visual field while was unreliable when stimuli were presented on the LVF (see Figure 1) and this is in keeping with Marzi et al.'s (1991) meta-analysis.

## Experiment 2

Experiment 2 used the same bimanual RT task employed in Experiment 1 (with anatomical and crossed hands) in a group of left-handed participants.

### Materials and methods

#### Participants

Twenty-eight students from the University of Bologna (11 females and 17 males, mean age = 21.15, SD = 1.97) participated in the experiment. They were all left-handed(−55/100, SD = 28.95) as assessed with the Edinburgh Handedness Inventory (Oldfield, 1971).

Apparatus, Stimuli, and procedure were the same as in Experiment 1. Response omissions (0.3%), unimanual responses (2.2%), responses faster than 120 ms (0.8%) and slower than 700 ms (0.2%) were discarded. Correct RTs were submitted to the same mixed ANOVA as in Experiment 1.

### Results

**Figure 2:**
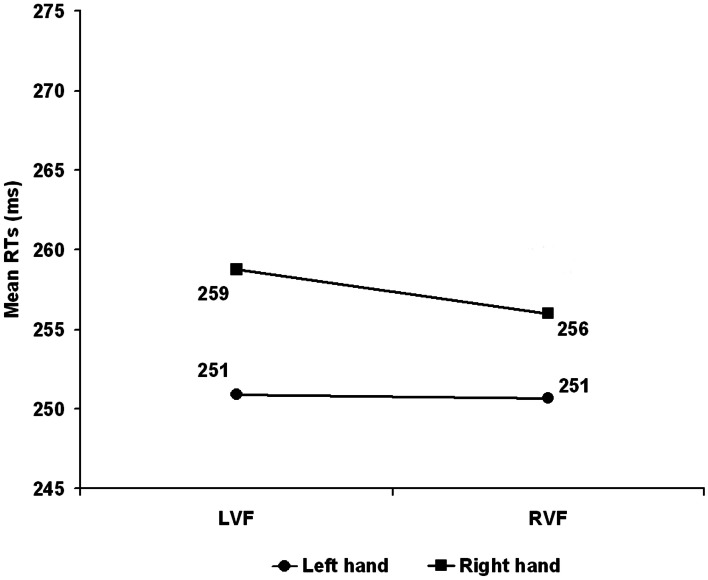
**Experiment 2: Left Handers**. Mean reaction time (RT) as a function of visual hemifield of stimulus presentation and response hand. LVF, Left visual field; RVF, right visual field.

The *Hand Arrangement* main effect was significant *F*_(1, 26)_ = 5.749, *p* = 0.024 with the anatomical slower than the inverted arrangement (265 vs. 243 ms). The *Visual Field* main effect was not significant (LVF = 255 vs. RVF = 253) *F*_(1, 26)_ < 1, *p* = 0.376 whereas the *Hand* main effect was significant with the dominant left hand faster (251 ms) than the right (257 ms) *F*_(1, 26)_ = 20.528, *p* < 0.001. The *Visual Field* × *Hand arrangement* interaction was just significant *F*_(1, 26)_ = 4.217, *p* = 0.050 with reliably faster RTs with inverted compared to anatomical arrangement for the LVF (243 vs. 267 ms) *t*_(26)_ = 2.850, *p* = 0.008 but not for the RVF (244 vs. 262 ms) *t*_(26)_ = 1.905, *p* = 0.068. The *Hand* × *Hand arrangement F*_(1, 26)_ < 1, *p* = 0.574 was not significant while, consistently with Experiment 1, the *Visual Field* × *Hand* interaction, witnessing the presence of an overall CUD of +1.5 ms, reached significance *F*_(1, 26)_ = 32.458, *p* < 0.001 with the dominant left hand faster than the right in both the LVF (251 vs. 259 ms) and the RVF (251 vs. 256 ms) but with a larger CUD in the LVF (see Figure 2).

More importantly, as in Experiment 1 this effect was independent from hand arrangement as demonstrated by the non-significant *Hand Arrangement* × *Visual Field* × *Hand* interaction *F*_(1, 26)_ = 2.077, *p* = 0.161.

Thus, in both right- and left-handers bimanual RTs with lateralized visual stimuli yielded a significant CUD which was not affected by spatial compatibility. This strengthens the hypothesis that anatomical factors, such as callosal transfer, are responsible for the slower responses to stimuli presented contralaterally to the responding hand.

## Discussion

This study has provided evidence supporting an “anatomical” explanation of the CUD effect in the Poffenberger paradigm with bimanual responding. The “anatomical” explanation posits that the CUD depends on a longer route involving callosal transmission during the crossed with respect to the uncrossed hemifield-hand condition. The crucial role of the CC has been established by behavioral studies in callosum sectioned or agenetic patients (Marzi et al., 1991; Zaidel and Iacoboni, 2003; Savazzi et al., 2007) or by a series of electrophysiological (Rugg et al., 1985; Marzi et al., 2003), transcranial magnetic stimulation (Marzi et al., 1998) and brain imaging studies (Marzi et al., 1999; Tettamanti et al., 2002; Omura et al., 2004; Weber et al., 2005; Mazerolle et al., 2008, 2010; Gawryluk et al., 2011). Moreover, a direct comparison of anatomical and spatial compatibility effects has been carried out by Anzola et al. (1977) and by Berlucchi et al. (1977) with a similar conclusion supporting the “anatomical” explanation. However, all the above studies employed a unimanual RT paradigm and in principle the relative importance of SRSC vs. anatomical effects might be different under bimanual conditions (see Di Stefano et al., 1980).

To answer this question, in the present study we employed a Poffenberger paradigm with bimanual responses and anatomical or inverted posture of the hands with respect to right and left response buttons. To ascertain the role of handedness we extended the study to a population of left-handers whose bimanual performance in a Poffenberger paradigm has never been tested and in whom the relative role of anatomical vs. spatial compatibility factors might be different from that of right-handers.

We found that in both right-handers and left-handers the crucial interaction between the CUD, as assessed by the first order Hand by Visual field interaction, and Hand arrangement was always far from significance thus ruling out a reliable effect of inverting the anatomical hand posture. Interestingly, Experiment 1 on right-handers confirmed a CUD asymmetry that was larger in the right than the left visual field thus confirming previous findings (see Marzi et al., 1991; Marzi, 2010). This asymmetry showed a tendency to be reversed in left-handers; a result that is also in keeping with previous evidence (Marzi et al., 1991).

Two further variables need to be tested for a thorough assessment of the role of anatomical vs. SRSC factors in the study of laterality effects in simple unimanual and bimanual RT, namely gender and hand posture in writing (in left-handers). These two variables could not be tested in the present study but in principle they might influence the weight of anatomical vs. SRSC factors in explaining the CUD.

### Conflict of interest statement

The authors declare that the research was conducted in the absence of any commercial or financial relationships that could be construed as a potential conflict of interest.

## Footnotes

^1^For both Experiment 1 and Experiment 2, the same ANOVA performed on RTs was also performed on variance. No sources of significance were observed (*F*s < 1) indicating a similar variance associated with crossed and uncrossed hemifield-hand conditions.
